# Bioenergetic Impairment of Triethylene Glycol Dimethacrylate- (TEGDMA-) Treated Dental Pulp Stem Cells (DPSCs) and Isolated Brain Mitochondria are Amended by Redox Compound Methylene Blue †

**DOI:** 10.3390/ma13163472

**Published:** 2020-08-06

**Authors:** Krisztina Mikulás, Timea Komlódi, Anna Földes, Gergely Sváb, Gergő Horváth, Ádám Miklós Nagy, Attila Ambrus, Szabolcs Gyulai-Gaál, István Gera, Péter Hermann, Gábor Varga, László Tretter

**Affiliations:** 1 Department of Prosthodontics, Semmelweis University, 1088 Budapest, Hungary; mikulas.krisztina@dent.semmelweis-univ.hu (K.M.); hermann.peter@dent.semmelweis-univ.hu (P.H.); 2 Department of Medical Biochemistry, MTA-SE, Laboratory of Neurobiochemistry, Semmelweis University, 1094 Budapest, Hungary; komlodi.timea@med.semmelweis-univ.hu (T.K.); svab.gergely@med.semmelweis-univ.hu (G.S.); horvath.gergo@med.semmelweis-univ.hu (G.H.); adamnagy@me.com (Á.M.N.); ambrus.attila@med.semmelweis-univ.hu (A.A.); 3 Oroboros Instruments, 6020 Innsbruck, Austria; 4 Department of Oral Biology, Semmelweis University, 1089 Budapest, Hungary; foldes.anna@dent.semmelweis-univ.hu (A.F.); varga.gabor@dent.semmelweis-univ.hu (G.V.); 5 Department of Oral Diagnostics, Semmelweis University, 1088 Budapest, Hungary; gyulai-gaal.szabolcs@dent.semmelweis-univ.hu; 6 Department of Periodontology, Semmelweis University, 1088 Budapest, Hungary; gera.istvan@dent.semmelweis-univ.hu

**Keywords:** DPSC, methylene blue, mitochondria, oxygen consumption, TEGDMA

## Abstract

Background: Triethylene glycol dimethacrylate (TEGDMA) monomers released from resin matrix are toxic to dental pulp cells, induce apoptosis, oxidative stress and decrease viability. Recently, mitochondrial complex I (CI) was identified as a potential target of TEGDMA. In isolated mitochondria supported by CI, substrates oxidation and ATP synthesis were inhibited, reactive oxygen species production was stimulated. Contrary to that, respiratory Complex II was not impaired by TEGDMA. The beneficial effects of electron carrier compound methylene blue (MB) are proven in many disease models where mitochondrial involvement has been detected. In the present study, the bioenergetic effects of MB on TEGDMA-treated isolated mitochondria and on human dental pulp stem cells (DPSC) were analyzed. Methods: Isolated mitochondria and DPSC were acutely exposed to low millimolar concentrations of TEGDMA and 2 μM concentration of MB. Mitochondrial and cellular respiration and glycolytic flux were measured by high resolution respirometry and by Seahorse XF extracellular analyzer. Mitochondrial membrane potential was measured fluorimetrically. Results: MB partially restored the mitochondrial oxidation, rescued membrane potential in isolated mitochondria and significantly increased the impaired cellular O_2_ consumption in the presence of TEGDMA. Conclusion: MB is able to protect against TEGDMA-induced CI damage, and might provide protective effects in resin monomer exposed cells.

## 1. Introduction

Research in dentistry, particularly in the conservative sector, has lately focused on the development of resin-based composites, which can provide preventive and aesthetic treatments, and can support both mechanical and chemical adhesion to the tooth surface [1]. Despite their obvious advantages, resin composites and adhesive systems may also display undesirable effects, such as cytotoxicity or genotoxicity; the underlying mechanisms are still rather obscure, but currently also hot topics in the research area [2,3,4,5,6,7].

The composite matrix consists of various viscous monomers, like urethane dimethacrylate (UDMA), 2-hydroxyethyl methacrylate (HEMA) and the co-monomer triethylene glycol dimethacrylate (TEGDMA). Due to its 50 wt% concentration and incomplete polymerization in composite restorative fillings, TEGDMA may readily leach out into the oral environment, including the mucosal and dental tissues [8,9,10,11,12,13,14]. Hydrolytic disintegration and the action of salivary esterases may lead to further monomer release. The monomers, released via any of the above mechanisms, at a distance of less than 1 mm, may readily reach the pulp in low millimolar concentrations, and penetrate the gingival epithelium and dentin [15,16,17]. TEGDMA is the main resin co-monomer released from composites [5,9,10,11], which can impair the function of neighboring cells, including dental pulp stem cells (DPSCs).

DPSCs play a crucial biological role in dental regenerative capacity [18,19,20,21], exhibit mesenchymal stem cell characteristics and serve to renew the dentin and the pulp following damage [22,23,24]. They also have marked anti-inflammatory effects, thereby counteracting local inflammation [25]. Therefore, their investigation in response to routinely applied agents in dentistry is most relevant for dental biocompatibility studies. Indeed, the direct effect of TEGDMA on DPSCs has been extensively studied, but the actual mechanism of action of this composite is not fully understood [26,27,28,29,30,31,32,33]. Understanding the patomechanism of TEGDMA is very important in order to interfere with its damaging effect during clinical applications.

TEGDMA resin monomers are toxic to dental pulp fibroblasts and DPSCs, can induce apoptosis, oxidative stress, deplete glutathione formation, decrease viability [5,34,35,36] and trigger genetic mutations [37,38,39,40,41,42].

Our current understanding is that oxidative stress and its downstream effects are the predominant pathomechanisms of the cytotoxicity of TEGDMA [5,43,44].

It was reported earlier that TEGDMA exhibited some of these deleterious effects via mitochondrial impairment [45,46,47,48,49].

In our previous report, using isolated mitochondria, we identified Complex I (CI) of the mitochondrial electron transport system, as a target of TEGDMA [48]. TEGDMA inhibited the CI-dependent O_2_ consumption, which resulted in the depolarization of the mitochondrial membrane. As a consequence of the latter, ATP production decreased; thereby, TEGDMA also caused bioenergetic insufficiency. Inhibition of CI by TEGDMA enhanced the mitochondrial hydrogen peroxide (H_2_O_2_) generation (H_2_O_2_ is one of the most stable forms of the reactive oxygen species [ROS]), and impaired the elimination of ROS; these two phenomena together led to the consequential induction of oxidative stress and apoptosis. The compromised ATP synthesis at high TEGDMA concentrations may also lead to necrotic cell death.

The effect of TEGDMA on human gingival fibroblasts resulted in dose-dependent ATP depletion and mitochondrial membrane potential (Δψ_m_) collapse, showing that mitochondria are involved in TEGDMA caused oxidative damage [46]. A previous study indicated that TEGDMA treatment suppressed the activity of Complex III (CIII) in mouse dental papilla cells [49]. TEGDMA released from resin composites stimulated the mitochondrial production of ROS and induced alterations in morphology of mitochondria [47].

Methylene blue is one of the oldest synthetic drug; it was first mentioned in biomedicine more than 120 years ago (Guttmann, P.; Ehrlich, P. Über die Wirkung des Methylenblau bei Malar. Berl. Klin. Wochenschr. 28: 953–956; 1891). Methylene blue has been used to treat CN poisoning [50], methemoglobinemia [51], cytostatics adverse effects [52]. Methylene blue (MB) proved to be beneficial in several neurological diseases [53] where various deficiencies of the mitochondrial CI, Complex II (CII) and/or CIII were detected. It was reported by us earlier that MB partially restored the mitochondrial respiration and ATP synthesis, as well as rescued the mitochondrial membrane potential in mitochondria treated with CI or CIII inhibitors [54]. These phenomena can be explained by the fact that MB is capable of providing an alternative pathway to the electron transfer from NADH [55] or FADH_2_ [56] towards cytochrome c [57] or molecular oxygen [54] *via* bypassing CI, CII and CIII. Moreover, MB also promotes an alternative mechanism of ATP synthesis, referred to as substrate level phosphorylation (SLP), in the tricarboxylic acid (TCA) cycle catalyzed by succinyl-CoA ligase [58]. 

The aim of this work was to investigate the potentially beneficial bioenergetic effects of MB on TEGDMA-treated mitochondria isolated from guinea pig brain cortex and on permeabilized or intact DPSCs. 

On the basis of the background information depicted above we hypothesized that the TEGDMA-mediated inhibition of CI function detected in isolated mitochondria should be detected in a cellular system as well. Both mitochondria and DPSCs were acutely exposed to millimolar concentrations of TEGDMA and a low concentration of MB which corresponds to the blood level of MB detected after iv. injection [59]. We used permeabilized DPSCs to investigate the effects of TEGDMA on the rate of oxygen consumption. Being successful in demonstrating the dominant inhibition of TEGDMA on the oxidation of CI substrates we performed experiments on intact DPSCs, and dose dependent decrease of mitochondrial oxygen consumption was detected. Applying MB to TEGDMA-treated mitochondria, improvement of bioenergetic functions was expected. Both in isolated mitochondria and in DPSCs MB stimulated the oxygen consumption in the presence of TEGDMA, in isolated mitochondria the TEGDMA-mediated depolarization of Δψ_m_ was partially restored by MB.

## 2. Materials and Methods

### 2.1. Chemicals

TEGDMA (triethylene glycol dimethacrylate) was obtained from VOCO, GmbH (Cuxhaven, Germany). The purity of TEGDMA was 97%; the remaining 3% was triethylene glycol monomethacrylate. The UV inhibitor content was 150 ppm (0.015%) HQME (hydroquinone monomethyl ether), which is common to a methacrylate monomer.

TEGDMA was dissolved in dimethyl sulfoxide (DMSO) and stored at −20°C. Standard laboratory chemicals were purchased from Sigma-Aldrich (St. Louis, MO, USA). Cell culture plates and calibration plates for Seahorse XF96 Analyzer, XF Plasma Membrane Permeabilizer were purchased from Agilent Technologies (Santa Clara, CA, USA). Methylene blue was purchased from Sigma-Aldrich.

### 2.2. Cell Isolation and Culturing

Impacted wisdom teeth were surgically removed from healthy young patients, aged between 17 and 30, at the Department of Oral Diagnostics, Semmelweis University. The procedure was performed obeying the guidelines of the Ethical Committee of the Hungarian Medical Research Council. This study was approved by the Semmelweis University Regional and Institutional Committee of Science and Research Ethics (permission number: 25459/2019/EKU). Dental pulp stem cells (DPSCs) were isolated according to our previous protocol [23], with some modifications. Briefly, the pulp tissue was mechanically chopped and then digested by collagenase (type I, Sigma-Aldrich, 1 mg/mL) in PBS for 1 h at 37 °C, using regular homogenization (at every 10 min). After centrifuging at 250× *g* for 5 min at 4 °C, the supernatant was removed, and the pelleted cells were seeded into a 10 cm cell culture plastic dish (Orange Scientific, Braine-l’Alleud, Belgium), containing culture medium MEM alpha (Lonza Group Ltd., Basel, Switzerland), supplemented with 10% FBS (Gibco/Invitrogen Corporation, Carlsbad, CA, US), 1% penicillin/streptomycin (Gibco/Invitrogen Corporation) and 1% glutamine (Gibco/Invitrogen Corporation). Cells were left growing at 37 °C under 5% CO_2_ and passaged using an 0.25% trypsin/EDTA solution upon 70% confluence (2–4 passages were permitted in the course of our experimentations).

### 2.3. Isolation of Mitochondria from Guinea Pig Brain Cortex

Mitochondria were isolated from guinea pig brain cortex using a Percoll gradient, as before [60,61]. Animals were housed and handled in accordance with the Guidelines for Animal Experiments of Semmelweis University. Briefly, after a rapid removal of the brain cortex, the sample was homogenized in Buffer A (in mM: 225 mannitol, 75 sucrose, 5 HEPES, 1 EGTA, pH 7.4) on ice, and centrifuged for 3 min at 1300× *g*. Afterwards, the supernatant was centrifuged for 10 min at 20,000× *g*, and the pellet was resuspended in 15% Percoll solution, and layered on a discontinuous Percoll gradient. After the differential centrifugation steps, the pellet was resuspended in Buffer B (in mM: 225 mannitol, 75 sucrose, 5 HEPES, pH 7.4). All the above operations were carried out either on ice or at 4 °C [62]. Mitochondrial protein concentration was determined using a modified Biuret method [63]. For our experiments, mitochondria were applied in 0.1 or 0.05 mg/mL final protein concentration. Results are expressed as μmol/min/mg protein O_2_ consumption.

### 2.4. Parallel Detection of Mitochondrial Oxygen Consumption and Membrane Potential (Δψ_m_)

Mitochondrial respiration was monitored using high-resolution respirometry Oroboros O2k (Oroboros O2k, Oroboros Instruments, Innsbruck, Austria). A two-point calibration for O_2_ concentration was applied, using zero O_2_ concentration (0%) and air saturation (100%). Mitochondrial membrane potential (Δψ_m_) was detected simultaneously with O_2_ consumption by Oroboros O2k equipped with the O2k-LED2 Fluo-Module, excitation LED 465 nm short pass filter and emission long pass filter, using fluorescent dye safranin O (2 µM).

### 2.5. Semiquantitative Assessment of Δψ_m_

Notably, Δψ_m_ was expressed applying a percentage (%) scale, where 100% (Fmin) corresponded to the membrane potential measured in the presence of respiratory substrate(s) and absence of ADP (LEAK respiration). At the end of each experiment, carbonyl cyanide-*4*-(trifluoromethoxy)phenylhydrazone (FCCP, an uncoupler, given in excess) fully depolarized the mitochondria (Fmax = 0%). Calculations for Δψ_m_ applied the following formula: Δψ_m_-related fluorescence (%) = 100 × (1 − (F(saf) − Fmin)/(Fmax − Fmin)) F(saf) is the actual safranin O fluorescence. At the beginning of the experiment, 2 µM safranin was added into the O2k-chamber. Afterwards, respiratory substrates pyruvate plus malate, glutamate plus malate or succinate (all in 5 mM concentration) were given to initiate LEAK respiration. Oxidative phosphorylation (OXPHOS) was initiated by the addition of ADP (2 mM), which was followed by administration of TEGDMA (5 mM) or DMSO (as control). Carboxyatractilozide (CAT, an inhibitor of the adenine nucleotide translocase [ANT], 2 µM) or oligomycin (Oligo), an inhibitor of the ATP synthase (2 µM) were titrated to reach LEAK-respiration once more. At the end of the experiment, FCCP (0.25 µM) was given to fully depolarize the mitochondria. All the above experiments were performed at 37 °C using the following respiratory medium (in mM): 125 KCl, 20 HEPES, 2 KH_2_PO_4_, 0.1 EGTA, 1 MgCl_2_, 0.025% BSA, pH 7.0.

### 2.6. Determination of Oxygen Consumption and Extracellular Acidification Rates 

Mitochondrial oxygen consumption rate (OCR) and extracellular acidification rate (ECAR, a representative parameter of the glycolytic flux) were measured using Seahorse XF96 Analyzer [64,65] (Agilent Technologies, Santa Clara, CA, USA), as previously described. DPSC were seeded 24 h prior to the assays at a 10^4^ cells/well density in 150 μL supplemented standard culture MEM alpha medium using Poly-*l*-lysine coated XF96 cell culture microplates (Agilent Technologies, Santa Clara, CA, USA). Moreover, 2 h prior to each measurement, the culture medium was exchanged to 180 μL XF assay medium. Alterations in O_2_ and H^+^ concentrations were detected, and thereafter, OCR and ECAR were calculated via the XF96 Analyzer software. Stock solutions for glucose (respiratory substrate, 10 mM), 2,4-dinitrophenol (DNP, uncoupler, 100 µM), TEGDMA (0.1, 0.5, 1, 2 or 5 mM), DMSO (solvent control), oligomycin (ATP synthase inhibitor, 2 µM), antimycin A (Complex III inhibitor, 1 μM) and rotenone (Complex I inhibitor, 1 µM) were all prepared in the assay medium and injected into the wells in the course of the respective experiments.

### 2.7. Cell Membrane Permeabilization 

For selective permeabilization of the cell membrane, the cells were treated with the XF Plasma Membrane Permeabilizer reagent (PMP, Agilent Technologies, Santa Clara, CA, USA, 1 nM), which was dissolved in the Mitochondrial Assay Solution (MAS, in mM: 660 mannitol, 210 sucrose, 30 KH_2_PO_4_, 1.5 MgCl_2_, 6 HEPES, 3 EGTA, 0.6% fatty acid free bovine serum albumin [BSA]), supplemented with 4 mM ADP. The optimal PMP concentration was determined using a different plate. Pyruvate (P, 10 mM) plus malate (M, 5 mM), alfa-ketoglutarate (α-KG, 10 mM), glutamate (G, 10 mM) plus malate (10 mM) or succinate (S, 10 mM), all dissolved in MAS, served as respiratory substrates, and were given into the wells prior to the respective experiments. TEGDMA (5 mM) or DMSO, CAT (2 µM), DNP (100 µM), and myxothiazol (Myx, Complex III inhibitor, 1 µM), all prepared in MAS, were also injected into the wells in the course of the respective measurements.

### 2.8. Statistical Analysis

Statistical analyses were performed using SigmaStat and/or GraphPad Prism 8.4.0. For testing the normal distribution, the Shapiro–Wilk test was applied. For multiple comparisons, the one-way ANOVA algorithm was applied, which was followed by Bonferroni’s post-hoc test. Simple Student’s t-test was applied for pair-wise comparison.

## 3. Results

### 3.1. Effect of TEGDMA and MB on the Mitochondrial Oxygen Consumption and Membrane Potential 

#### 3.1.1. Oxygen Consumption of Isolated Brain Mitochondria

**Figure 1:**
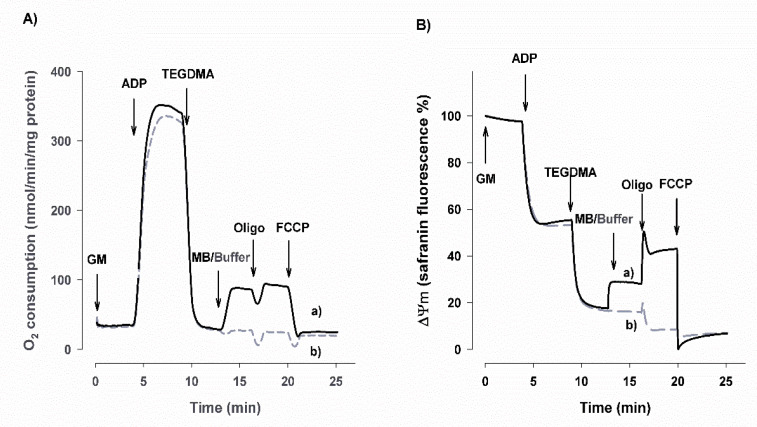
Effects of TEGDMA on the oxygen consumption (**A**) and membrane potential (Δψ_m_; **B**) of isolated brain mitochondria in the presence or absence of methylene blue (MB; 2 µM), respiring on glutamate plus malate (GM). Mitochondria were incubated in the ’respiratory medium’ described under Materials and methods. ADP, TEGDMA and MB (black curve), TEGDMA and Buffer (dashed gray curve), oligomycin (Oligo), and uncoupler (FCCP) were given as indicated on the original representative traces. Semiquantitative estimation of Δψ_m_. On the basis of safranin, fluorescence was calculated as detailed in Methods, according to the following equation: Δψ_m_ (%) = 100 × (1 − (F(saf) − Fmin)/(Fmax − Fmin)).

Guinea pig brain mitochondria were supported by CI substrates glutamate plus malate (GM) or pyruvate plus malate (PM), and ADP. The ADP-stimulated respiration was inhibited by TEGDMA (5 mM) in similar degrees: by 92% for GM and PM, respectively (Table 1 and Table 2, Figure 1A). In succinate-supported mitochondria, TEGDMA did not exert a significant inhibitory effect on the ADP-stimulated respiration (data not shown). It is known that selected redox compounds can accept electrons from various members of the electron transport system (ETS), and can even be reoxidized by other compounds of the ETS. Previously, it was shown that inhibition of the mitochondrial CI by rotenone can be bridged by methylene blue (MB) [54]. It is shown in Figure 1 that TEGDMA (5 mM), given after ADP, exerted a powerful and very fast inhibitory effect on the respiration of GM-supported mitochondria. This inhibition could be ameliorated by MB (Table 1 and Figure 1 for GM substrates and Table 2. for PM substrates). MB added after TEGDMA more than doubled the rate of oxidation (from 30.6 ± 2.7 to 85.6 ± 2.8 and from 31.4 ± 3.2 to 75.8 ± 3.6) (fourth columns of Table 1 and Table 2). However, the rate of respiration in the presence of both TEGDMA and MB is still much lower than those of the untreated controls (first columns of Table 1 and Table 2). In TEGDMA-treated mitochondria, addition of oligomycin decreased the rate of respiration, however, only minimally. In TEGDMA plus MB treated mitochondria, addition of oligomycin did not significantly change the O_2_ consumption (Table 1 and Table 2). Addition of an uncoupler dissipated the Δψ_m_ and stimulated O_2_ consumption in both the untreated and MB-treated controls. Contrary to that, in the presence of TEGDMA, addition of the uncoupler decreased the rate of oxidation (not shown). 

#### 3.1.2. Alterations in Δψ_m_ in the Presence of TEGDMA and MB

Although O_2_ consumption is a valuable parameter in the assessment of mitochondrial functions, several potential mechanisms might be found behind any alteration (decrease, increase or staying steady) in it. To uncover these mechanisms, complementary mitochondrial parameters must be determined. Among them, one of the most important parameters is the mitochondrial membrane potential. 

In parallel with the O_2_ consumption, safranin fluorescence, an indicator of the Δψ_m_, was also monitored (Figure 1B, and Table 3). It is important to note that the method applied in these experiments is semiquantitative (it is taken as 100% when only the respiratory substrate is present, while the fluorescence detected after the addition of excess uncoupler is considered to be 0% membrane potential). Between 0 and 100%, a linear scale was considered and applied. In the experimental paradigm described above, the addition of ADP resulted in a depolarization. The most important statistically significant results are: (i) adding 5 mM TEGDMA almost entirely depolarized the mitochondria (~90% decrease; x_1_, x_2_). In case of the TEGDMA-treated mitochondria, oligomycin further depolarized Δψ_m_ (near to 0%; Table 3 (x_3_) and Figure 1B). Applying MB after TEGDMA polarized mitochondria by 10% from 10 to 19%, respectively, significance is indicated by (#). This polarization was further enhanced by oligomycin, from 19% to 36.6%, (§).

In the following stage, studies were extended towards more complex cellular systems. Using permeabilized cells, it was possible to validate results obtained using isolated mitochondria, and understand additional results originating from studies using intact cells.

### 3.2. Effects of TEGDMA on the Oxygen Consumption of Permeabilized DPSCs

**Figure 2:**
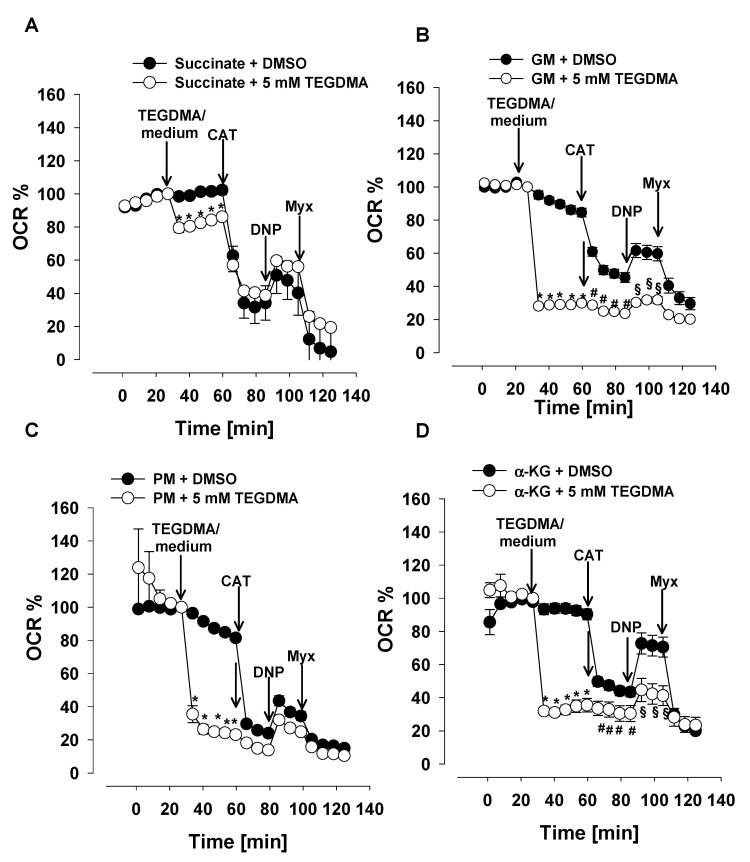
Relative oxygen consumption of permeabilized DPSC respiring on succinate (**A**), glutamate plus malate (**B**), pyruvate plus malate (**C**) and α-ketoglutarate (**D**). Cells were permeabilized prior to the experiment, as described under Materials and Methods. Respiratory substrates and ADP (4 mM) were added to the permeabilized cells before the assay. TEGDMA (5 mM), carboxyatractilozide (CAT; 2 µM), 2,4- dinitrophenol (DNP; 100 µM) and myxothiazol (Myx; 1 µM) were injected as indicated. Each point shows mean values of 16–36 parallel wells ± SEM. OCR (oxygen consumption rate) is expressed as %. The last measurement points before TEGDMA or DMSO injection is considered to be 100%. *, #, § indicate significant differences (*p* < 0.05) between TEGDMA-treated and control cells after the TEGDMA, CAT, or DNP additions, respectively.

To identify what mitochondrial pathways are affected by TEGDMA, DPSCs’ plasma membrane was permeabilized and mitochondria were energized by either NADH-linked (CI-linked; PM, GM, α-KG) substrates or by CII substrate succinate. The presence of ADP enabled the oxidative phosphorylation (Figure 2). As it is seen in Figure 2, TEGDMA significantly decreased the O_2_ consumption in cells respiring on PM (from 84.9 ± 0.8 to 24.3 ± 1.8%), GM (from 86.2 ± 2.5 to 28.9 ± 1.3%), and α-KG (from 92.5 ± 3 to 34.9 ± 3.7%) (Figure 2B–D). However, in cells energized with succinate, the respiration was decreased only by 16% (from 101.5 ± 2.2 to 84.2 ± 2.3%; Figure 2A). The effect of TEGDMA on succinate-supported mitochondria was also significant.

Addition of CAT, an inhibitor of adenine nucleotide translocase (ANT), led to further decrease in the O_2_ consumption regardless of the respiratory substrates used. However, when CAT was added to TEGDMA-treated mitochondria, the diminution of the O_2_ consumption by CAT was smaller as compared to that observed in the control cells respiring on either GM, PM or α-KG. In mitochondria supported by succinate, the CAT-induced decreases in the O_2_ consumption in percentages were nearly identical in the presence and absence of TEGDMA. 

To detect maximal respiration, the uncoupler DNP was injected, which dissociated the O_2_ consumption from ATP synthesis in the mitochondria. The maximal respiration was higher in the absence of TEGDMA in two out of three CI substrate supported mitochondria (Figure 2B,D), but was not significantly different in mitochondria supported by succinate, or PM (Figure 2A,C). Each experiment was ceased by the Complex III inhibitor myxothiazol (Myx), a compound that almost entirely inhibits O_2_ consumption.

### 3.3. Oxygen Consumption in Dental Pulp Stem Cells (DPSCs)

**Figure 3:**
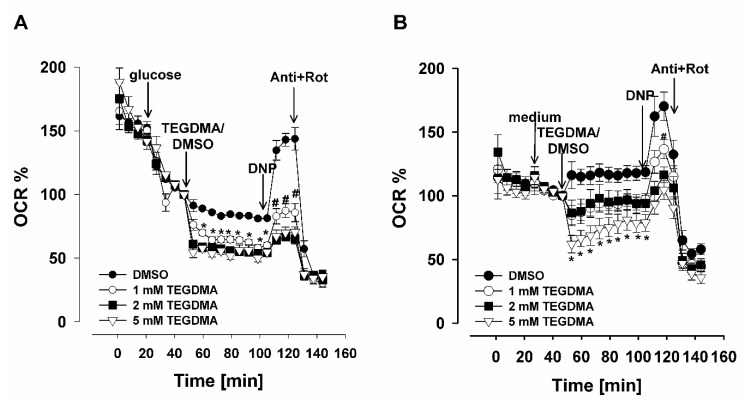
Relative oxygen consumption of dental pulp stem cells in the presence (**A**) and absence of glucose (**B**). Glucose (10 mM; A) or XF medium (**B**), TEGDMA (1; 2 or 5 mM) or DMSO, 2,4-dinitrophenol (DNP; 100 µM) and antimycin A (Anti; 1 µM) plus rotenone (Rot; 1 µM) were injected as indicated. Each symbol represents different concentrations of TEGDMA. OCR (oxygen consumption rate) is expressed as %. The last data point before TEGDMA or DMSO injection is 100%. Each point shows mean values of 18-38 parallel wells ± SEM. Statistics: * indicates significant difference (*p* < 0.05) after TEGDMA addition. For panel A) OCR at TEGDMA 1, 2, and 5 mM concentrations, for panel B) OCR at 5 mM TEGDMA concentration differs from control. # indicates significant difference in the OCR after DNP addition. Both in A) and B) OCR-s at all TEGDMA concentration were different from that of DMSO-treated DPSCs.

The effects of TEGDMA on O_2_ consumption (basal respiration) was also tested in adherent DPSCs. To promote glycolysis, glucose was injected, while in the other set of experiments, XF respiration medium was applied to support the oxidative metabolism in the cells (Figure 3). Various concentrations of TEGDMA (1, 2 and 5 mM) were applied, to study its effect on respiration of living (non-permeabilized) cells. When glycolysis was activated by glucose, in the presence of 1 mM, 2 mM and 5 mM TEGDMA, respectively, the O_2_ consumption was decreased to 64.6 ± 2.5%,(1 mM TEGDMA), 55.9 ± 2.4% (2 mM TEGDMA) and 51.9 ± 2.9% (5 mM TEGDMA). Afterwards, the uncoupler dinitrophenol (DNP) was injected to reach the maximal respiration. In control cells, in the absence of TEGDMA, the DNP-evoked maximal respiration was 143 ± 5 %; in cells treated with TEGDMA (5 mM) the maximal respiration only reached 70 ± 4.3%, at time = 105–117 min, when glucose was also present (Figure 3A). In cells not energized by glucose, the maximal respiration after DNP addition was also lower after a 5 mM TEGDMA treatment (104.4 ± 74%), as compared to the control (170.3 ± 11.2%; at time = 105–117 min) (Figure 3B). Each protocol was terminated by the addition of the CIII inhibitor antimycin A (Anti), as well as CI inhibitor rotenone (Rot), to inhibit O_2_ consumption and detect non-mitochondrial O_2_ consumption.

### 3.4. Effects of TEGDMA on the Rate of Extracellular Acidification by DPSCs

**Figure 4:**
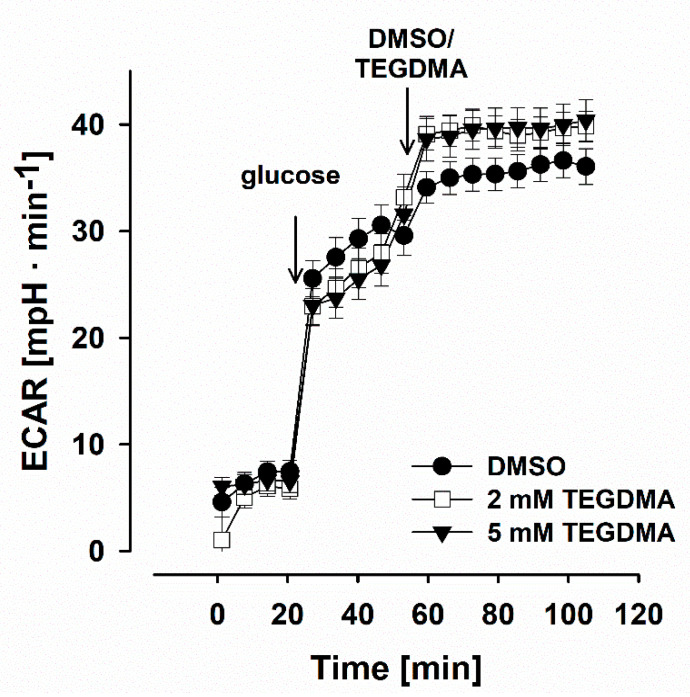
Effect of TEGDMA on extracellular acidification rate (ECAR) of dental pulp stem cells in the presence of glucose. Glucose (10 mM), TEGDMA (2 or 5 mM) or DMSO were injected as indicated. Each symbol represents different concentrations of TEGDMA. Data are expressed as [mpH·min^−1^]. Each point shows mean values of 28–39 parallel wells ± SEM. No significant differences were observed between groups.

The cellular O_2_ consumption and extracellular acidification rate (ECAR) were detected simultaneously. ECAR is the alteration in the extracellular pH over time, and considered to be an indirect marker of glycolysis [66]. Glucose injection led to an increase in ECAR, which implies an augmented glycolysis (Figure 4). Injection of TEGDMA (2 and 5 mM) resulted in a small, non-significant elevation in ECAR (from 35 ± 2 mpH·min^−1^ to 40 ± 2 mpH·min^−1^ at 5 mM TEGDMA). 

### 3.5. Effects of MB on the Oxygen Consumption of TEGDMA-Treated DPSCs

**Figure 5:**
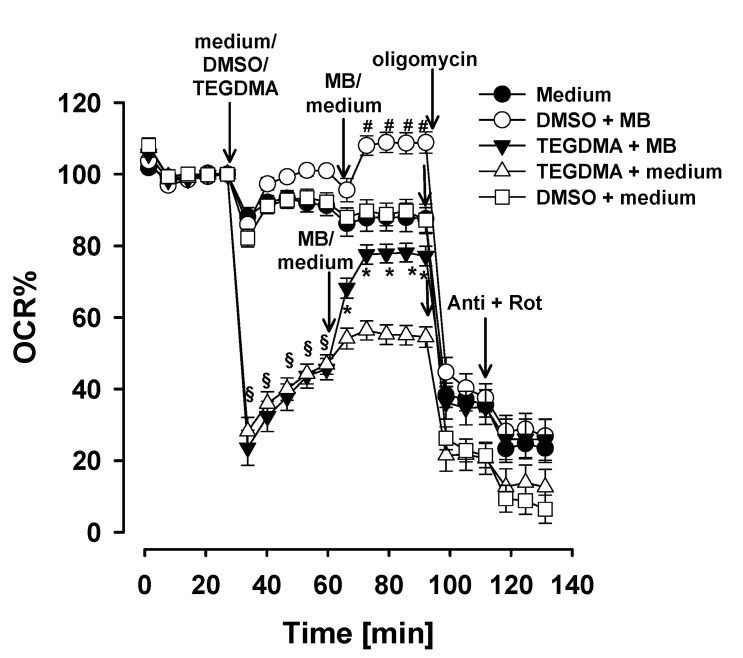
Effect of methylene blue (MB) on oxygen consumption of TEGDMA-treated dental pulp stem cells. DMSO, XF medium or TEGDMA (5 mM), MB (2 µM), oligomycin (2 µM), antimycin A (Anti; 1 µM) and rotenone (Rot; 1 µM) were injected as is indicated. OCR (oxygen consumption rate) is expressed as %. The last data point before TEGDMA or DMSO or medium injection is considered to be 100%. Each point shows mean values of 39–65 parallel wells ± SEM. *N* = 8. Statistics: § indicates difference between TEGDMA and DMSO+medium *p* < 0.001. * indicates difference between TEGDMA and TEGDMA + MB *p* < 0.001; # indicates difference between DMSO + medium and DMSO + MB *p* < 0.001.

As observed in permeabilized cells, TEGDMA exerted an inhibitory effect on the NADH-linked pathway, supported by either PM, GM or α-KG, while the succinate-linked pathway was much less affected. This implies an inhibition by TEGDMA in the electron transfer system (ETS) upstream of the CIII, which leads to an accumulation of the reducing equivalent NADH. Therefore, we tested the neuroprotective compound MB like with isolated brain mitochondria, which is able to take over electrons (getting reduced) from NADH (and FADH_2_) and can transport them to cytochrome c in the case of CI or CIII inhibition as well [54,55,57]. First, TEGDMA (5 mM), DMSO or XF medium was injected to the DPSC cells (Figure 5). As was seen in Figure 5, TEGDMA led to a decrease in the OCR relative to the control (from 100 to 25.8 ± 2.3%). The addition of MB significantly increased the respiration when TEGDMA was also present (from 45.6 ± 3 to 77.1 ± 2.8%, *p* < 0.001). The treatment of cells with MB in the absence of TEGDMA significantly stimulated resting respiration relative to the control (DMSO or XF medium). Next, oligomycin, an inhibitor of the F_1_F_O_-ATP-synthase, was added, which decreased the O_2_ consumption relative to the control (DMSO or XF medium). When both TEGDMA and MB were injected into the cells, the inhibitory effect of oligomycin was smaller in magnitude (20.7 ± 4.5%), as compared to the control (when MB was omitted, 35 ± 4.9%). The protocols were again terminated by simultaneous additions of Antimycin plus Rotenone, to detect non-mitochondrial oxidation.

## 4. Discussion

One of the most important properties of dental adhesives and composite filling materials is that the biocompatibility has become a clinically relevant issue [7]. Different citotoxicity tests of seven latest dental adhesives containing methacrylate monomers (HEMA, UDMA, Bis-GMA, TEGDMA, glycerol phosphate dimetacrylate-(GPDMA)) and hydrophilic monomers (4-methacryloyloloxyethy trimellitate anhydride-(4-META), methacryloyloyloxyethyl trimellitic acid-(4MET), methacryloyloxydecyl dihydrogenphosphate-(MDP)thiouracil monomer-(MTU-6)) were investigated on human gingival fibroblast by Pagano et al. Their results show that citotoxicity differed according to the dental adhesive was investigated [67].

It was reported that TEGDMA adversely affects cellular metabolism, signaling, differentiation, and dentinogenesis, while it also interferes with wound healing and tissue repair [28,40,68,69,70,71]; it induces oxidative stress [46,47,48], apoptosis and/or necrosis [42,43,49,72]. Due to its amphiphilic character, TEGDMA can penetrate the membranes of human gingival fibroblast and human oral epithelium of the mouth, resulting in an inflammatory response [73]. Furthermore, TEGDMA triggers time- and concentration-dependent toxicity in mouse gingival fibroblasts and dental papilla mesenchymal cells in three-dimensional cultures [71]. 

DPSCs play a crucial biological role for the regenerative capacity of the dental pulp [19,20], in pulp repair processes. Deep caries or restorative procedures affect destruction of the odontoblastic layer or release of xenobiotics into the pulp [18,21]. DPSCs are able to have a protective mechanism toward resinous monomers by their autocrine signaling, excluding at higher monomer concentrations, which can lead to irreversible pulp damage [32] and by retaining their stemness and self-renewal capacity [31]. DPSCs were elected for this study because they are well characterized stem cells that can contribute a reliable indication of the compounds’ biocompatibility [19]. 

Implication of mitochondrial bioenergetics as target of the damaging effects of TEGDMA started with the papers of Schmalz et al. and Lefeuvre et al. [46,74] Numerous studies indicate that the phenotiazin derivative MB has a wide range of positive biological effects. The aim of the present study was to extend the bioenergetical approach of investigation of TEGDMA from isolated mitochondria to permeabilized DPSCs and to intact DPSCs, and to elucidate the possible beneficial effects of the neuroprotective redox compound MB on isolated mitochondria, and on TEGDMA-treated DPSCs.

### 4.1. Effects of TEGDMA and MB on Bioenergetics of Isolated Mitochondria

It has been observed that TEGDMA inhibits mitochondrial O_2_ consumption at the CI level [48]. In line with that study, our present results confirm the mitochondrial targets of TEGDMA, mainly the vulnerability of CI. Using CI substrates, TEGDMA (5 mM) inhibited the ADP-stimulated oxidation of these substrates by 92% (Table 1 and Table 2); TEGDMA-evoked decrease of oxygen consumption can be partially corrected by MB (Figure 1A, Table 1 and Table 2). Measurement of mitochondrial membrane potential revealed that TEGDMA also decreased the Δψ_m_ in the presence of CI substrates. Due to the inhibition of CI by TEGDMA, electrons cannot flow freely, the proton pumping activity will be inhibited, and consequently Δψ_m_ will be depolarized (Figure 1B and Table 3). Addition of MB elevated Δψ_m_, i.e., polarized the mitochondria taking electrons from flavoproteins and from the reducing equivalents then transferring them downstream of the block most probably to the cytochrome c component of the electron transfer system [57]. This bypass, referred to as “alternative electron transfer” [57,75] permits the flow of electrons and increases the proton pumping activity of the CIV (cytochrome oxidase), thus both the O_2_ consumption and Δψ_m_ get augmented. Addition of the ATP synthase inhibitor oligomycin to “untreated” mitochondria can decrease OCR almost back to the value of LEAK respiration (Table 1 and Table 2 first column). In the present study, oligomycin displayed differing effects under control conditions and in TEGDMA-treated mitochondria, in the absence or presence of MB. Under control conditions, oligomycin blocked the backflow of protons from the intermembrane space towards the matrix. The membrane became hyperpolarized and this slowed down the proton pumping activity and electron flow (Table 2 and Table 3). In the presence of TEGDMA, the rate of ATP production exhibited a dose-dependent decrease. However, ATP that was produced in the presence of the respiratory substrate(s) and ADP, before the addition of TEGDMA, may re-enter into the mitochondria and can be hydrolysed by the ATP synthase working in its “reverse mode”. Under these conditions, the ATP synthase pumped protons out into the intermembrane space and thus contributed to the build-up process of Δψ_m_. Blocking the ATP synthase by oligomycin blocked both the forward and reverse modes, therefore blocking the proton pumping activity of the ATP synthase triggered a collapse in Δψ_m_. (Figure 1B and Table 3). In the presence of MB, the blockage at CI was partially relieved, the electrons from MB were transferred to cytochrome c and then to CIV. CIV possesses a proton-pumping activity (two H^+^ *per* two e^−^), thus not only the electron flow and O_2_ consumption was accelerated (Figure 1A, Table 1 and Table 2), but the Δψ_m_ also became hyperpolarized (Figure 1B and Table 3). Addition of oligomycin, under these conditions, further increased the Δψ_m_. To summarize: in the presence of TEGDMA, mitochondria became depolarized and exhibited a low rate of O_2_ consumption, whereas, in the presence of TEGDMA and MB, a higher rate of oxidation was associated with a higher Δψ_m_. Afterwards, the uncoupler FCCP was added to fully dissipate Δψ_m_. 

### 4.2. The Effects of TEGDMA on Mitochondrial Substrate Oxidation of Permeabilized DPSC-s 

In order to extend investigations from isolated organelles to more complex systems, permeabilized cells represented an intermediate step. Studies on permeabilized DPSC affirmed our earlier observation, that oxidation of CI substrates (a-KG, pyruvate plus malate and glutamate plus malate) is sensitive to TEGDMA-mediated inhibition (Figure 2B–D). Contrary to that the results obtained in isolated mitochondria [48], in succinate supported permeabilized cells a small but statistically significant inhibition of oxygen consumption was observed (Figure 2A). A possible explanation of this controversy could be the incomplete permeabilization of the cell membrane and the consequent imperfect wash out of CI substrates which could “contaminate” the oxidation of succinate via their high sensitivity to TEGDMA-mediated inhibition.

These results (the inhibition of CI) have relevance to ROS homeostasis. It has been described that inhibition of CI is accompanied by excessive ROS production which can induce cell damage and apoptosis in a dose-dependent manner [76,77]. In line with these observations, Liu et al. revealed that 72 h TEGDMA exposure gave rise to cell death and increased ROS formation in human gingival fibroblasts [47]. Furthermore, in a mouse preodontoblast cell line mDPC6T, TEGDMA decreased cell viability, and induced excessive ROS formation [49]. 

### 4.3. Effect of TEGDMA on Oxygen Consumption of DPSCs

Measurement of O_2_ consumption of living cells is one of the most sensitive indicators of mitochondrial function. In living, non-permeabilized cells, the basal respiration is dependent on the cellular energy demand, energy metabolism and mitochondrial coupling [66]. Under this condition the O_2_ consumption relies on either the substrates in the culture media or on the endogenous substrate supply. The effects of TEGDMA on the oxygen consumption rate were investigated on DPSCs supported by endogenous substrates or by glucose. TEGDMA applied in the range of 1–5 mM showed a dose-dependent inhibition of O_2_ consumption both in the presence and absence of glucose. Both the basal respiration and the uncoupler-induced maximal respiration were inhibited by TEGDMA. 5 mM TEGDMA is near to the highest possible TEGDMA concentration present in the pulpa in vivo after methacrylates leach out into the oral environment [9,11,78]. In line with our data, Bakopoulou and her group recently published that TEGDMA triggered concentration-dependent cytotoxicity in DPSCs, followed by cell cycle arrest at high concentrations of TEGDMA [28]. 

### 4.4. TEGMA Does Not Affect Glycolytic Flux

To investigate the effect of TEGDMA on the glycolytic pathway, the extracellular acidification rate (ECAR) was simultaneously determined with the oxygen consumption on DPSCs. ECAR, the alteration of pH over time, is considered as an indirect marker of the glycolytic activity *via* lactate production and the corresponding acidification of the medium [66]. However, it should be emphasized that there are also other sources of protons, such as CO_2_ release from the TCA cycle, which cannot be excluded under this condition, either [66]. It was observed that in glucose supported, glycolytically active cells, TEGDMA (5 mM) did not inhibit ECAR, but even a small-scale statistically not significant stimulation was detected (Figure 4). Since the mitochondrial O_2_ consumption and ATP synthesis are coupled, inhibition of O_2_ consumption is accompanied by a reduced ATP synthesis. Therefore, regulatory mechanisms could stimulate glycolysis to provide ATP for the cells *via* fermentation. To shed light on the direct effect of TEGDMA on glycolysis, further experimental work (e.g. measurement of glycolytic enzyme activities) would be required. 

### 4.5. Effect of Methylene Blue on TEGDMA-Treated and Untreated DPSCs

In the present study the possible beneficial effects of MB on TEGDMA-treated, non-permeabilized DPSCs were also investigated. Similarly to that observed in isolated brain mitochondria (Figure 1A, Table 1 and Table 2), in DPSCs MB significantly increased O_2_ consumption in the presence of TEGDMA as compared to TEGDMA-treated cells (Figure 5). At the 80th min of the experiment OCR of TEGMA-treated cells was 55.2 ± 2.7%, in the presence of TEGDMA plus MB OCR was higher: 77.8 ± 2.6%. Applying MB to control cells generated a similar, about 20% increase in OCR. This latter phenomenon might be explained by the fact that MB-evoked alternative electron transport (bypassing respiratory complexes) could decrease the efficacy of proton pumping in the ETS, therefore the ATP production per O_2_ consumption ratio (P/O ratio) will be decreased and the decrease of [ATP] will stimulate respiration [54].

### 4.6. TEGDMA, MB, Mitochondria and ROS Production

Many researchers share the view that oxidative stress and its downstream effects are responsible for the cytotoxicity of TEGDMA [5,43,44]. Could we say that TEGDMA produces ROS, ROS will attack many structures and functions, among them mitochondria? Will ROS inhibit Complex I and is that the phenomenon investigated in some studies? The time-scale of experiments presented here do not support this scenario. In this study the effects of TEGMA were investigated on isolated mitochondria, permeabilized cells and intact cells. Applying TEGDMA to isolated mitochondria the effects were immediate. Using permeabilized cells and intact cells the timescale was similar. When TEGDMA was injected to the wells, the first datapoints were taken after a 3 min of mixing period. Observing the data (Figure 2, Figure 3 and Figure 5) we can conclude that the inhibitory effect of TEGDMA reached at least 90% of its maximum within 5 min after the injection. This very fast effect can be explained by a direct immediate inhibition on the complex I. Inhibition of the respiratory chain will result also in an immediate increase of ROS production and a decrease of ROS elimination, therefore oxidative stress will develop. 

The current study did not address the effect of MB on the TEGDMA-stimulated mitochondrial ROS production. The role of MB in the ROS homeostasis is controversial. In cellular studies, under various conditions, contradictory results were published. In many cellular systems, administration of MB decreased the ROS production [57,79]; for review see [53]. Contrary to this, in our previous report MB stimulated ROS generation and inhibited ROS elimination in mitochondria [54]. This controversy is resolved by Stack et al. 2014 [80] describing that MB has powerful effects on gene expression as well. It is able to activate the Nrf2-antioxidant element signaling pathway, therefore stimulate the antioxidant defense [80] The effects of MB are predominantly pro-oxidant in isolated cell organelles, whereas they can be antioxidant in cellular systems depending upon conditions. Besides that, MB is frequently used in antimicrobal photodynamic therapy [81] to kill bacteria. To judge the effects of MB on various cellular processes we have to take into account the hormetic dose-response curve of MB [52] meaning that it can exhibit opposing effects depending upon the dose of treatment.

### 4.7. Limitations of the Study 

The results presented here are limited to isolated mitochondria and to DPSC under acute conditions. Further investigations (in vivo as well) are required to reveal the long-term effects of MB or related compounds on TEGDMA-induced cell toxicity, apoptosis, mutagenesis and oxidative stress, and to efficiently decrease the harmful side effects of dental resins.

## 5. Conclusions

We conclude that TEGDMA inhibits the mitochondrial O_2_ consumption supported by Complex I substrates in isolated mitochondria and in permeabilized DPSCs. TEGDMA decreases O_2_ consumption in intact DPSCs as well, without exerting an obvious inhibition on the glycolytic flux. MB, a redox cycler, is able to amend the TEGDMA-inhibited mitochondrial O_2_ consumption, and the drop off membrane potential in isolated mitochondria. MB is also able to increase oxygen consumption of TEGDMA-treated DPSCs. 

It would be intriguing to investigate the long-term effects of MB and its non-coloured analogues on TEGDMA-exposed cells and TEGDMA-triggered oxidative stress, cytotoxicity, and inflammation in particular. As DPSCs are the cellular sources of tissue repair and key components of the anti-inflammatory regulation during dental pulp damage, therefore protecting them against TEGDMA-induced damage may have direct clinical relevance. 

## Figures and Tables

**Table 1:** Effects of TEGDMA on oxygen consumption of brain mitochondria supported by glutamate plus malate in the presence or absence of methylene blue.

	UNTREATED n = 5	+TEGDMA n = 7	+MB n = 5	TEGDMA Plus MB n = 7
GM	36.5 ± 1.5	33.2 ± 1.5	37.9 ± 2.5	31.6 ± 1.4
ADP	285.8 ± 17.6	330 ± 13.5 (x_1_)	290.6 ± 12.9	313.5 ± 14.9 (x_2_)
TEGDMA	-	27.6 ± 1.7 (x_1_)	-	30.6 ± 2.7 (x_2_) (#)
MB	-	-	272.2 ± 10	85.6 ± 2.8 (#)
Oligo	40.7 ± 2.4	23.3 ± 2.1 (*)	78.1 ± 1.7	89.2 ± 3.9 (*)

Glutamate plus malate (GM 5-5 mM); ADP (2 mM) TEGDMA (5 mM); methylene blue (MB 2 µM); oligomycin (Oligo 2 µM) were added as indicated in the Materials and methods section and are depicted on the Figure 1A. Oxygen consumption is expressed in µmol/min/mg protein. Results are expressed as mean ± S.E.M. n indicates the number of measurements. Corresponding statistical difference pairs are indicated. *p*(x_1_) < 0.001; *p*(x_2_) < 0.001; *p*(#)< 0.001; *p*(*) < 0.001.

**Table 2:** Effects of TEGDMA on oxygen consumption of brain mitochondria supported by pyruvate plus malate, in the presence or absence of methylene blue.

	UNTREATED n = 7	+TEGDMA n = 5	+MB n = 5	TEGDMA Plus MB n = 5
PM	30.9 ± 4.2	36.7 ± 0.4	30.8 ± 3.7	35.3 ± 2.5
ADP	265.3 ± 28.7	308.2 ± 24.6 (x_1_)	269.6 ± 17	311.8 ± 18.8 (x_2_)
TEGDMA	-	25.8 ± 1.5 (x_1_)	-	31.4 ± 3.2 (x_2_) (#)
MB	-	-	269.4 ± 20.1	75.8 ± 3.6 (#)
Oligo	41.3 ± 4	21.8 ± 1.4 (*)	65.4 ± 4	82.6 ± 5.7 (*)

Pyruvate plus malate (PM 5-5 mM); ADP (2 mM) TEGDMA (5 mM); methylene blue (MB 2 µM); oligomycin (Oligo 2 µM) were added as indicated in the Methods section, and are depicted in Figure 1A. Oxygen consumption is expressed in µmol/min/mg protein. Results are expressed as mean ± S.E.M. n indicates the number of measurements. Corresponding statistical difference pairs are indicated. *p*(x_1_) < 0.001; *p*(x_2_) < 0.001; *p*(#) < 0.001; *p*(*) < 0.001.

**Table 3:** Effects of TEGDMA on mitochondrial membrane potential of brain mitochondria supported by glutamate plus malate (GM), in the presence or absence of methylene blue (MB).

	+TEGDMA n = 7	TEGDMA Plus MB n = 7
GM	100%	100%
ADP	48.2 ± 2.2 (x_1_)	46.8 ± 2.7 (x_2_)
TEGDMA	10.2 ± 1.7 (x_1_) (x_3_)	10.1 ± 2.3 (x_2_) (#)
MB	-	19.1 ± 2.7 (#), (§)
Oligo	3.8 ± 0.9 (*) (x_3_)	36.6 ± 4.1 (*), (§)
FCCP	0%	0%

Mitochondria were incubated in the respiratory medium. Glutamate plus malate (GM 5-5 mM); ADP (2 mM); TEGDMA (5 mM); methylene blue (MB 2 μM); oligomycin (Oligo 2 μM) were added as indicated in the Methods section, and are depicted on the Figure 1B. Percentage of membrane potential was calculated as described in the Materials and Methods section. 100% indicates hyperpolarization, 0% indicates complete depolarization in the presence of uncoupler (FCCP). Results are expressed as mean ± S.E.M. n indicates the number of measurements. Corresponding statistical difference pairs are indicated. *p*(x_1_) < 0.001; *p*(x_2_) < 0.001; *p*(x_3_) 9 < 0.001; *p*(#) < 0.05; *p*(*) < 0.001, *p*(§) < 0.001.
